# Enhancing Suicide Prevention Knowledge and Self-Efficacy Among Public School Educators in Rajasthan, India: an implementation study from Thar desert district of India

**DOI:** 10.1192/j.eurpsy.2026.11982

**Published:** 2026-07-31

**Authors:** N. Rustagi, N. Suthar", N. Nebhinani, S. Sharma, I. Arora, N. Dahiya, A. Grover, P. Raghav

**Affiliations:** 1 Community Medicine; 2 Psychiatry, AIIMS Jodhpur, Jodhpur; 3 Divison of Implementation Research, ICMR, Delhi, India

## Abstract

**Introduction:**

Suicide prevention training for educators is critical for early identification and intervention with at-risk youth.

**Objectives:**

To evaluates the effectiveness of a **Gatekeeper Training (**GKT) program towards suicide prevention awareness and self-efficacy among **Public School Educators** educators in Rajasthan, India.

**Methods:**

The current study was planned as part of National Health related priority project funded by Indian council of Medical Research. An implementation study was carried out for government schools for Rajasthan, India where educators were invited to engage in gatekeeper training for enhancing suicide prevention knowledge and their perceived self-efficacy in improving student’s mental wellbeing. Quantitative outcomes included knowledge/awareness and self-efficacy scales, analysed with paired and independent t-tests and effect size calculations (Cohen’s d). Thematic analysis was conducted for open ended discussions captured throughout the sessions.

**Results:**

A total of 126 educators from urban Jodhpur schools participated (50.8% female; mean age = 43.8 years, mean experience = 16.7 years), Significant improvements were observed in both awareness (mean increase = 1.04, Cohen’s d = 0.615, p < 0.001) and self-efficacy (mean increase = 1.16, Cohen’s d = 0.524, p < 0.001), both reflecting medium effect sizes. The strongest gains were reported for identifying warning signs (d = 0.806, large effect) among at risk students. Male educators showed significantly higher post-intervention awareness (p = 0.024), self-efficacy (p = 0.001), and intervention acceptability (p = 0.040) than females. Implementation measures (acceptability, appropriateness, feasibility) scored high (≥85% of maximum). Qualitative themes revealed improved understanding of suicide as a process, enhanced empathy, and myth-busting of common misconceptions.

**Conclusions:**

The gate keeper training program was well-received, feasible, and effective for public funded school educators. Male educators reported somewhat higher post-training gains as comapred to female educators. Thematic analysis highlighted increased sensitivity to warning signs and the importance of empathetic, private dialogue with at risk students realised among educators after training sessions.

**Disclosure of Interest:**

None Declared

